# Prof. Armor—Prize Essay

**Published:** 1853-09

**Authors:** 


### PROF. ARMOR—PRIZE ESSAY.

We learn, though not officially, that Prof. Armor, formerly Professor of Physiology and Pathology in the "College of Physicians and Surgeons of the Iowa University," has been recently appointed to the same position in the "Ohio Medical College," at Cincinnati. Prof. Armor is a ripe medical scholar and lecturer, and a most efficient teacher. During his connection with us, we admired him for his profound science; we loved him for his amenity of manner and his courteous bearing towards his colleagues and the classes, and we honored him for his strict integrity, his unimpeached, and unimpeachable, moral character. He is an ornament to the profession, and an honor to his species; and while we breathe a silent prayer for his future success in life, there is mingled in the same cordial aspiration, the anxious wish that in the profession there were more like him, and that the world at large were but made up of such.

It is with no ordinary feelings of pride and pleasure that we announce to the profession and the personal friends of Prof. Armor, that in the contention for the prize recently in Ohio, he came off triumphant. One hundred and twenty-five dollars were offered as an award for the best Medical Prize Essay! The contest was a warm one, and highly interesting; but the committee, after carefully examining the several essays, gave the prize to Prof. Armor. The subject was the Zymotic theory of essential fevers: and it is a highly creditable and interesting production. We shall endeavor to make room for portions of it, in a future number.

